# Multimodal imaging in conductive hearing loss: Optimising CT, MRI and CTA for accurate diagnosis and management

**DOI:** 10.4102/sajr.v30i1.3278

**Published:** 2026-02-12

**Authors:** Athanasios Vlachodimitropoulos, Michail Athanasopoulos, Afroditi Lepida, Pinelopi Samara, Ioannis E. Papachristos, Theodoros Stathas, Spyridon Lygeros, Georgios Batsaouras

**Affiliations:** 1 Department of Otolaryngology, University Hospital of Patras, Patras, Greece; 2 Department of Radiology, Primary Health Care Center of Agios Alexios, Patras, Greece; 3 Children’s Oncology Unit ‘Marianna V. Vardinoyannis-ELPIDA’, Aghia Sophia Children’s Hospital, Athens, Greece; 4 Department of Diagnostic Radiology, Biotypos, Chalandri, Greece

**Keywords:** conductive hearing loss, HRCT, MRI, CTA, ossicular chain, cholesteatoma, otosclerosis

## Abstract

Conductive hearing loss (CHL) results from impaired mechanical transmission of sound through the external or middle ear and is commonly associated with conditions such as otosclerosis, cholesteatoma, ossicular discontinuity, congenital malformations and trauma. Less frequently, it may arise from vascular or neoplastic lesions. Imaging plays a central role in the evaluation of CHL, with high-resolution CT (HRCT) regarded as the gold standard for initial assessment because of its superior spatial resolution in detecting bony pathologies, including fenestral otosclerosis, ossicular chain defects, third-window lesions and trauma-related disruptions. MRI complements HRCT by providing excellent soft tissue contrast, facilitating the detection of postoperative cholesteatoma via non-echo-planar diffusion-weighted imaging and the characterisation of vascular tumours such as glomus tympanicum. CT angiography (CTA), while not routinely indicated, can be valuable for identifying vascular anomalies and aiding surgical planning in selected cases. Technical considerations, such as optimised scan parameters, strategies for radiation dose reduction in paediatric patients and the integration of multiple imaging modalities, are essential for accurate diagnosis and effective treatment planning. Collectively, HRCT, MRI and CTA provide a structured, evidence-based framework for the comprehensive evaluation and management of CHL.

**Contribution:**

This review synthesises current evidence on HRCT, MRI and CTA in the assessment of CHL, emphasising their complementary roles, protocol optimisation and multimodal integration to enhance diagnostic accuracy and surgical guidance in both paediatric and adult populations.

## Introduction

Conductive hearing loss (CHL) occurs when sound transmission through the external or middle ear is impaired, resulting in an air–bone gap on pure-tone audiometry. Unlike sensorineural hearing loss, which reflects cochlear or neural pathology, CHL is typically mechanical in origin. The major aetiologies include otosclerosis (characterised by stapes fixation because of aberrant otic capsule remodelling), chronic otitis media with or without cholesteatoma, ossicular chain discontinuity or congenital malformations and middle ear trauma. Less commonly, neoplastic or vascular lesions, such as paragangliomas, may be implicated.^1^ Additionally, inner-ear ‘third-window’ phenomena, particularly superior semicircular canal (SSC) dehiscence, can produce a conductive component by creating an abnormal pathway for sound energy dissipation.^2^

High-resolution CT (HRCT) and MRI are the cornerstone imaging tools for CHL. CT offers excellent depiction of the ossicular chain and bony labyrinth, whereas MRI provides complementary soft-tissue and vascular detail.^3^ CT angiography (CTA) has a more specialised role, primarily in delineating vascular architecture and aiding in the diagnosis of vascular middle ear lesions.^4^ This review synthesises current evidence on the diagnostic performance, technical protocols and clinical applications of CT, MRI and CTA in both adult and paediatric CHL. It also proposes tailored protocol recommendations and a comparative modality table to support a structured, evidence-based approach to evaluation and diagnosis.

## Unmasking conductive hearing loss with high-resolution CT

**FIGURE 1:**
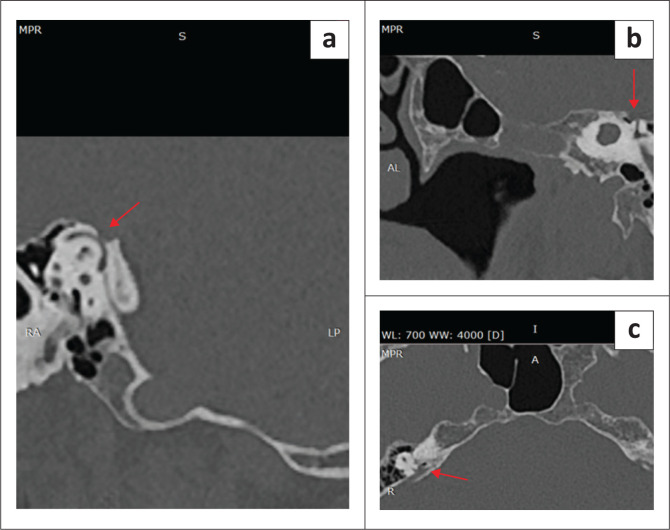
Superior semicircular canal dehiscence in a 35-year-old female patient. (a) Reformatted CT image in the Pöschl plane, clearly demonstrating the bony defect over the superior semicircular canal (red arrow). (b) High-resolution coronal reconstruction displaying the superior semicircular canal perpendicular to the long axis of the temporal pyramid (red arrow). (c) Stenvers plane reconstruction, oblique to the Pöschl plane and parallel to the long axis of the pyramid (red arrow).

High-resolution CT of the temporal bone is the first-line imaging modality for unexplained CHL when otoscopic and microscopic examinations are inconclusive.^3^ Using submillimetre isotropic voxels, HRCT provides micrometric delineation of the external auditory canal (EAC), tympanic cavity, ossicular chain, mastoid air cell system, facial nerve canal, jugular bulb and osseous labyrinth. This precision enables identification of fenestral otosclerosis, such as radiolucent foci at the fissula ante fenestram and round window niche obliteration.^5^ The technique also detects ossicular erosions, congenital fusions or dysplasias and occult traumatic injuries – including non-displaced fractures, incudostapedial joint dislocation or ligamentous avulsion. Tailored Pöschl and Stenvers reconstructions allow visualisation of SSC dehiscence (Figure 1) and other third-window lesions, while defects of the tegmen tympani or antrum, with associated meningocele or cerebrospinal fluid leak, are readily identified. In the study by Belden et al., SSC dehiscence was detected in all 36 patients with the clinical syndrome, including bilateral involvement in six patients; six additional patients without clinical signs appeared dehiscent on 1.0 mm CT, but intact bone was confirmed on 0.5 mm scans.^6^

High-resolution CT further characterises mastoid air cell pneumatisation patterns, which inform surgical planning and influence postoperative ventilation outcomes. Postoperatively, HRCT confirms stapes piston or ossicular prosthesis placement and assesses footplate fenestration following stapedotomy or ossiculoplasty.^7^

In well-aerated middle ear and mastoid spaces with an intact ossicular chain, HRCT demonstrates high negative predictive value for excluding occult cholesteatoma, granulation tissue or hypervascular tumours, thereby guiding clinical decision-making towards surveillance or surgical intervention only when indicated. In the study by Uz Zaman et al., HRCT exhibited a sensitivity of 100%, specificity of 88.1%, positive predictive value of 92.1% and negative predictive value of 100% for detecting cholesteatoma.^8^ Finally, advances in photon-counting and ultra-high-resolution detector technologies promise faster acquisition, reduced radiation dose and enhanced depiction of osseous detail, reinforcing HRCT as the benchmark modality for temporal bone imaging.^9^

### Decoding otosclerosis, cholesteatoma and congenital anomalies

**FIGURE 2:**
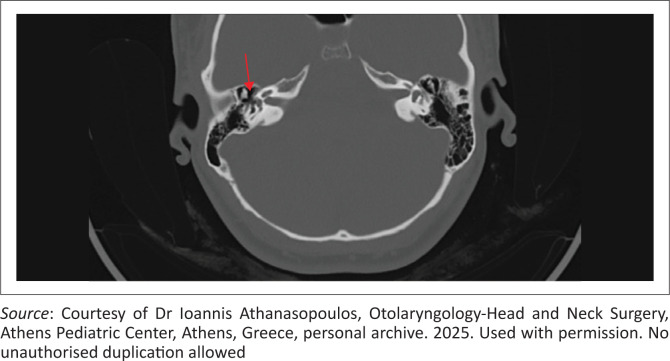
Axial CT image of the temporal bone in a 24-year-old female patient with otosclerosis, showing a hypodense focus in the otic capsule anterior to the oval window (fissula ante fenestram, red arrow), characteristic of fenestral otosclerosis. This lesion leads to conductive hearing loss by fixation of the stapes footplate.

Fenestral otosclerosis on sub-1 mm HRCT appears as a circumscribed hypodense focus anterior to the stapes footplate at the fissula ante fenestram – an imaging hallmark of active disease (Figure 2). Modern ultra-HRCT can detect lesions as small as 1 mm and frequently demonstrates asymptomatic contralateral involvement.^5^ Retrofenestral extension produces smooth pericochlear lucency, yielding the characteristic ‘double-ring’ or ‘halo’ sign. Recognition of this pattern, together with application of the Symons–Fanning CT grading system, refines disease staging and surgical planning. Nevertheless, dense sclerotic foci or early inactive lesions may fall below the resolution threshold, resulting in so-called ‘infra-radiological’ disease; thus, a normal HRCT cannot definitively exclude otosclerosis.^10^

**FIGURE 3:**
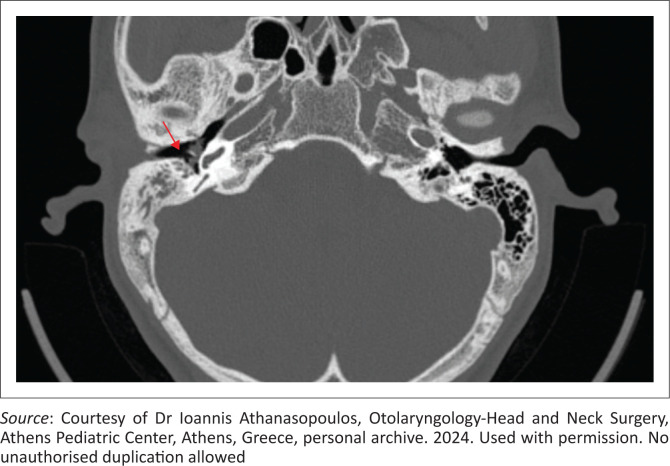
Axial CT image of the temporal bone in a 15-year-old male patient with right-sided cholesteatoma, showing soft tissue opacification and erosive changes of the ossicular chain (long process of the incus, red arrow), contributing to conductive hearing loss.

**FIGURE 4:**
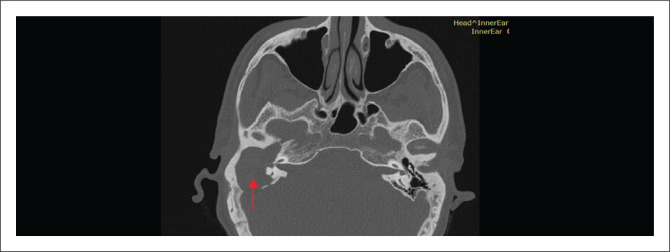
Right ear cholesteatoma in a 29-year-old male patient. Axial CT image demonstrating extensive bone erosions involving the labyrinthine capsule, ossicular chain and mastoid antrum walls (red arrow). Findings are consistent with advanced cholesteatoma.

In chronic suppurative otitis media, HRCT delineates the full extent of acquired cholesteatoma (Figure 3 and Figure 4), typically revealing nonspecific middle ear soft tissue associated with erosions of the scutum, ossicles, tegmen or labyrinth – findings considered pathognomonic.^8^ A well-aerated tympano-mastoid cavity with intact ossicles confers a negative predictive value approaching 100%, whereas soft tissue opacification without osseous erosion remains nonspecific. In such cases, diffusion-weighted MRI improves specificity when cholesteatoma cannot be excluded.^11^ Preoperative HRCT also identifies anatomic variants such as a low-lying tegmen, dehiscent sigmoid sinus or high-riding jugular bulb, all of which may alter the surgical approach.

Following temporal bone trauma, HRCT is unsurpassed in detecting subtle ossicular chain injuries, including incudostapedial and incudomalleolar separations, incus subluxation, malleolar handle fractures, stapes crura fractures and associated haemotympanum – all of which contribute to CHL and guide ossiculoplasty.^12^

**FIGURE 5:**
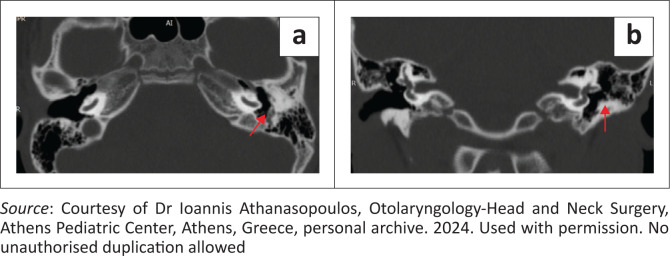
Left external auditory canal (EAC) atresia in a 7-year-old male patient. (a) Axial CT image demonstrates complete bony atresia of the left EAC with an absent canal lumen and a small, dysplastic middle ear cavity (red arrow). (b) Coronal CT image further shows the atretic EAC (red arrow).

Congenital ossicular anomalies and EAC atresia (Figure 5) – frequent causes of paediatric CHL – are also accurately characterised by HRCT. Submillimetre sections reveal absent or dysplastic ossicles, a fixed or monopodal stapes footplate, a narrowed or aplastic tympanic cavity and an aberrant facial nerve canal. Such detail is indispensable for counselling and for determining surgical feasibility.^13^ High-resolution CT-based aural atresia grading correlates with postoperative air-bone gap closure and assists in stratifying candidates for canalplasty or ossiculoplasty. Although MRI more effectively depicts cochlear-vestibular malformations and cochlear nerve aplasia, HRCT remains the skeletal roadmap of choice in congenital CHL.

High-resolution CT further detects rare vascular and neoplastic mimics. Tympanic paragangliomas appear as enhancing retrotympanic soft-tissue masses with moth-eaten enlargement of the jugular foramen, whereas an aberrant internal carotid artery presents as an intratympanic tubular structure coursing through a dehiscent or absent carotid canal – critical red flags prior to otologic surgery. When necessary, CT or MR angiography further delineates vascular supply, intracranial extension and cervical dissemination.^14^

### Paget’s disease: Beyond the basics of conductive hearing loss

Paget’s disease of bone involves the skull in approximately one-third of patients and, when affecting the petrous temporal bone, can distort sound-conducting structures, resulting in progressive mixed or purely CHL.^15^ High-resolution CT demonstrates cortical thickening, coarse trabeculation and lytic–sclerotic ‘cotton wool’ changes. Quantitative CT and histopathology further reveal otic capsule demineralisation and microfracturing, producing a diffuse third-window shunt responsible for low-frequency air–bone gaps despite an intact ossicular chain.^2^ Pagetic bone may also narrow the EAC, ankylose the ossicles or fracture the stapes footplate. Temporal bone studies confirm these lesions and support the role of exploratory ossiculoplasty or stapedotomy in carefully selected cases. Ultra-HRCT serves as the primary modality for staging and surgical planning, whereas MRI is reserved for atypical presentations to exclude marrow infiltration or sarcomatous transformation. Surgical intervention is best undertaken after biochemical remission and should be guided by updated CT to minimise the risks associated with drilling hypervascular, brittle bone.^16^

### Technical considerations: Which parameters matter?

Contemporary multidetector CT scanners routinely generate 0.5-mm isotropic datasets within a narrow field of view, reconstructed using high-spatial-frequency bone kernels to maximise cortical–cancellous contrast. Newer photon-counting platforms achieve 0.25-mm section thickness on a 2048 × 2048 matrix, enabling near-microscopic depiction of the stapes footplate, its crura and other minute middle ear structures. Axial acquisitions, obtained parallel to the infraorbital–meatal line or the lateral semicircular canal, are automatically reformatted into coronal and sagittal planes.^3^ Additional ultrathin oblique reformats aligned with the stapes footplate enhance detection of early fenestral otosclerosis that may be overlooked on standard orientations. Intravenous iodinated contrast is rarely required, as non-contrast studies best preserve osseous detail.

### CT: A powerful tool in expert hands

High-resolution CT is the cornerstone modality for evaluating CHL when osseous disease is suspected.^17^ Although highly effective at depicting bony lesions, its diagnostic sensitivity is stage dependent. In otosclerosis, workstation-based studies employing 0.5-mm collimation and multiplanar reconstructions detected CT abnormalities in 74% – 85% of surgically confirmed ears.^18^ Conversely, a 2017 systematic review and meta-analysis^19^ of 1078 ears reported a pooled sensitivity of only 58%, but a specificity of 95% underlining the persistence of ‘infra-radiological’ early lesions. When HRCT does reveal the classic fissula ante fenestram lucency or a pericochlear halo, its positive predictive value exceeds 90%.

Performance is generally more robust for cholesteatoma: in a cohort of 296 ears, sinus tympani disease was detected with 91% sensitivity and a 90% negative predictive value, and HRCT reliably demonstrated scutum blunting, ossicular lysis, tegmen dehiscence and labyrinthine fistula.^20^ A normally aerated epitympanum with intact ossicles on CT confers a high negative predictive value, often excluding occult cholesteatoma.^21^ However, CT cannot consistently distinguish cholesteatoma from effusion, granulation tissue or fibrosis, limiting specificity in the presence of soft tissue.^22^

Subtle ossicular chain injuries may sometimes only be confirmed intraoperatively, whereas temporal bone fractures are well visualised on HRCT. The modality also enables fracture classification (otic capsule-sparing vs. violating or longitudinal vs. transverse), which informs hearing prognosis.^23^

In summary, CT remains the gold standard for CHL because of its unparalleled ability to depict osseous abnormalities. When applied judiciously, particularly in paediatric populations where radiation exposure must be minimised, it provides essential diagnostic and preoperative information across the spectrum of CHL aetiologies.

## MRI as a high-fidelity diagnostic tool for conductive hearing loss

MRI is not typically the first-line investigation for pure CHL, as its spatial resolution is insufficient to depict the minute bony landmarks of the ossicular chain or the oval- and round-window complex. It becomes indispensable, however, when a soft-tissue process is suspected, the full extent of disease must be mapped, or a mixed sensorineural component is present. MRI’s superior soft-tissue contrast allows direct visualisation of fluid, inflammation, fibrosis and enhancement patterns that CT cannot demonstrate.^2^

**FIGURE 6:**
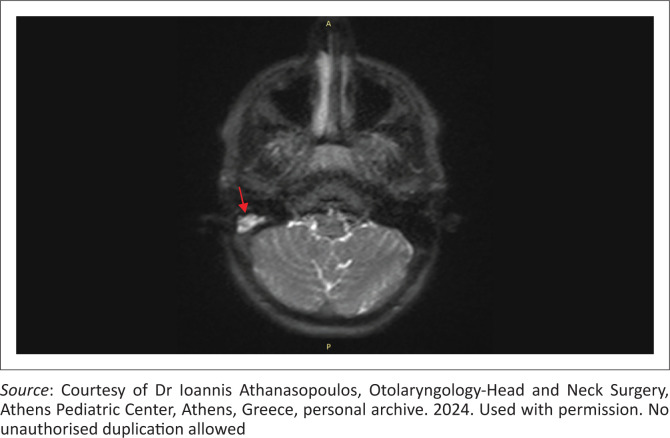
Axial non-echo-planar diffusion-weighted MRI of a 15-year-old male patient, confirming restricted diffusion of keratin debris within the cholesteatoma (red arrow), improving specificity in cases where CT alone is insufficient.

**FIGURE 7:**
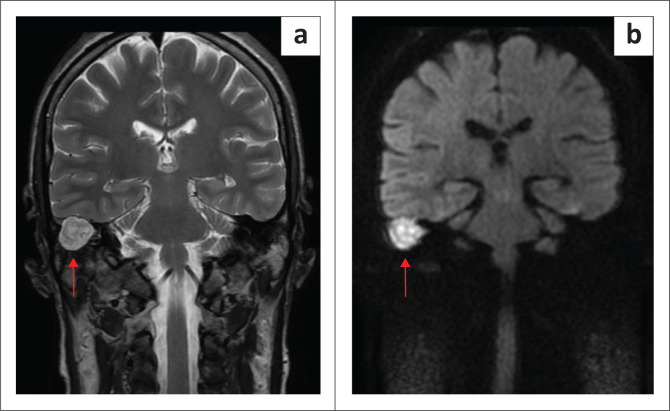
Right ear cholesteatoma in a 26-year-old female patient. (a) Coronal T2-weighted MRI and (b) coronal non-echo planar diffusion-weighted MRI images demonstrate the lesion, which shows increased T2 signal intensity and corresponding restricted diffusion consistent with cholesteatoma (red arrows).

In CHL, MRI is generally reserved for suspected cholesteatoma (Figure 6 and Figure 7) or middle ear tumours. For cholesteatoma – especially in complex or postoperative cases – dedicated sequences confirm residual disease and differentiate it from fibrosis. Non-echo-planar DWI has revolutionised evaluation by detecting restricted diffusion of keratin debris; cholesteatomas appear hyperintense with low apparent diffusion coefficient (ADC) values, enabling detection of foci as small as 2 mm – 3 mm.^24^ This non-invasive method often obviates the need for second-look surgery, as negative non-echo-planar DWI findings can reliably exclude residual or recurrent disease and allow patients to be followed radiologically rather than undergoing re-exploration.^25,26^

**FIGURE 8:**
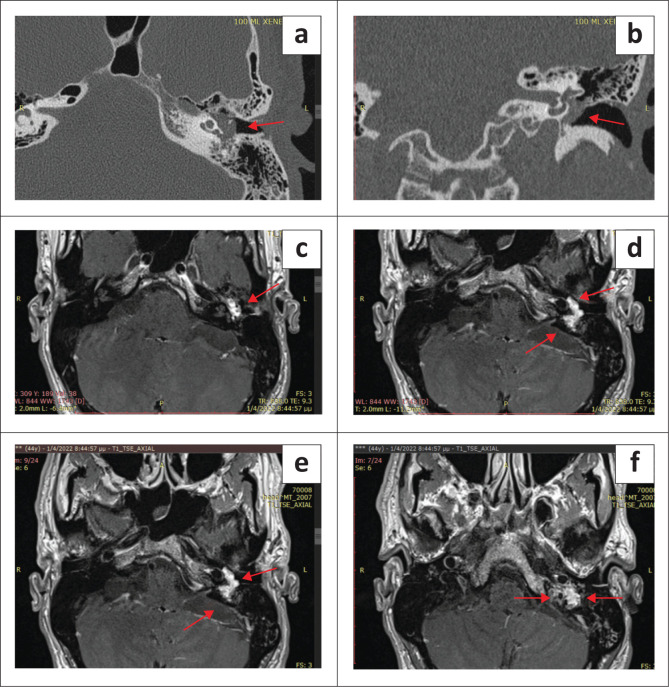
Glomus jugulotympanicum in a 44-year-old female patient. (a,b) Axial and coronal CT images demonstrating the lesion involving the jugular bulb and middle ear, classified as Grade 1 according to Glasscock–Jackson. (c,d,e,f)Contrast-enhanced MRI images of the same patient demonstrating the soft tissue extent of the intense contrast-enhancing tumour, highlighting its vascular nature (red arrows).

Combined with CT, MRI also reveals inner ear or intracranial complications – such as labyrinthitis, abscess or meningeal enhancement – that are crucial for surgical planning. Contrast-enhanced MRI is preferred for glomus tympanicum and jugulare tumours (Figure 8). These lesions typically appear as lobulated, avidly enhancing masses engulfing ossicles and vessels, exhibiting the characteristic ‘salt-and-pepper’ T1 pattern.^14^ Dynamic post-gadolinium imaging shows rapid arterial filling with early washout, differentiating paraganglioma from schwannoma or meningioma. Fat-suppressed post-contrast sequences delineate tumour extension from the hypotympanum into the jugular bulb, superiorly along the sigmoid sinus, medially into the petrous carotid canal and inferiorly into the carotid space. MRI further depicts marrow invasion, venous sinus thrombosis, extradural intracranial extension and perineural spread – findings essential for staging, selecting the surgical corridor and planning preoperative embolisation. Although HRCT remains unrivalled for cortical bone evaluation, MRI more accurately defines tumour margins, vessel encasement and soft-tissue infiltration, thereby guiding multidisciplinary management and reducing operative morbidity.^27^

MRI is also indicated in mixed hearing loss (conductive and sensorineural components) to evaluate the cochlea, vestibulocochlear nerve and brain for underlying pathology, such as cochlear otosclerosis, cochlear nerve deficiency or retro-cochlear lesions. Active otosclerotic disease extending to the cochlea may sometimes be appreciated on MRI, demonstrating enhancement of the otic capsule or cochlear inflammation, although CT remains the first-line modality for otosclerosis.^28^

In paediatric CHL, MRI complements CT by revealing inner ear malformations – such as cochlear aplasia or an enlarged vestibular aqueduct – and by confirming cochlear nerve presence and calibre, a critical consideration for cochlear implantation. While most of these malformations primarily cause sensorineural hearing loss, mixed patterns may occur, making MRI an important adjunct. For purely conductive congenital anomalies, MRI mainly serves to rule out syndromic inner ear abnormalities, whereas CT remains superior for evaluating ossicles and middle ear anatomy.^29^

### MRI reserved for middle ear mass or intracranial extension

Current recommendations support MRI as a second-line or complementary modality in CHL.^30^ When an initial CT identifies a soft tissue mass in the middle ear or mastoid, MRI is often indicated as the next step. According to the Appropriateness Criteria,^31^ contrast-enhanced MRI is ‘usually appropriate’ when a middle ear mass is suspected to represent a neoplasm or to extend into the intracranial compartment or inner ear. MRI provides superior delineation of lesion extent, demonstrating dural involvement, sigmoid sinus thrombosis, brain abscess or labyrinth invasion that may be incompletely depicted on CT. Preoperative imaging benefits from the complementary strengths of both modalities: CT outlines bony anatomy and erosions, whereas MRI maps soft tissue extent and distinguishes granulation tissue, cholesteatoma or tumour, together yielding a complete surgical roadmap. However, MRI is not recommended as the initial study for CHL in the absence of neurological symptoms or soft-tissue warning signs, as it offers limited additional value for isolated ossicular disease and may overlook findings that CT would readily detect.

### Technical considerations and optimisation of temporal-bone MRI protocol

Temporal-bone MRI begins with thin-section (≤ 1 mm) isotropic 3D heavily T2-weighted sequences – most commonly CISS, FIESTA-C or SPACE – to delineate labyrinthine and middle ear anatomy with high spatial resolution. Targeted axial and coronal 3D T2-FSE or CISS slabs are then acquired through the otic capsules, followed by axial non-echo-planar DWI to screen for cholesteatoma. Pre- and post-gadolinium T1-weighted sequences encompass the temporal bones and, when disease extension is suspected, are expanded to include the entire brain. If a vascular anomaly is suspected, 3D time-of-flight or contrast-enhanced MR angiography is incorporated. All sequences are obtained with submillimetre voxels using dedicated head or surface coils and a small field of view, as conventional thicker-slice brain MRI may overlook subtle lesions. Finally, the MRI dataset is co-registered with HRCT – or reviewed side by side – so that soft tissue findings correspond precisely to bony landmarks, optimising surgical planning.^32^

MRI examinations are longer (typically 30 min – 45 min) and may require patient cooperation or sedation in young children – a key consideration in paediatric CHL. Although MRI does not involve ionising radiation, the need for gadolinium contrast should be weighed carefully. For cholesteatoma specifically, non-contrast DWI alone is highly accurate, and adding gadolinium does not significantly improve the detection of cholesteatoma pearls. On the other hand, when a tumour is suspected, gadolinium-enhanced MRI is essential for lesion characterisation.

### Diagnostic performance

For cholesteatoma, non-EPI DWI MRI offers excellent diagnostic performance. Individual series report 80% – 100% sensitivity and specificity. A 2013 meta-analysis pooled both metrics at approximately 94%, and more recent meta-analyses from 2017 and 2023–2024 confirm even higher pooled values (around 96% sensitivity and 93% specificity), highlighting the incremental advantages of non-EPI techniques. Nevertheless, lesions smaller than 2 mm – 3 mm may evade detection, and false-negative results are more common during early postoperative surveillance.^33^ This technique is particularly valuable for confirming cholesteatoma in postoperative ears where CT may be confounded by scarring.

For paragangliomas, MRI is highly sensitive – approaching 100% for larger glomus tumours – showing both the lesion and its internal flow voids.^27^ Contrast-enhanced 3D time-of-flight MRA (CE-3D-TOF MRA) detects small temporal bone paragangliomas more accurately than routine contrast-enhanced MRI.^34^ In the landmark study by Neves et al., CE-3D-TOF MRA achieved 100% sensitivity and 94% specificity, whereas fat-suppressed contrast-enhanced T1-weighted imaging reached only 94% sensitivity and 41% specificity.^35^ Larger head-and-neck series report pooled values of approximately 89% sensitivity and 99% specificity when digital subtraction angiography is used as the reference standard.^36^

## CT angiography in conductive hearing loss – from CT to CT angiography: Mapping vascular details

CT angiography is a specialised form of CT that visualises blood vessels following intravenous contrast administration. In the evaluation of CHL, CTA is not routinely indicated and is generally not part of first-line imaging unless specific findings suggest its necessity. The American College of Radiology guidelines note that there is no evidence supporting routine CTA for uncomplicated CHL. However, CTA becomes highly useful when a vascular anomaly or tumour is suspected as the underlying cause of hearing loss.

A classic example is a middle ear paraganglioma (glomus tympanicum or glomus jugulare). While MRI can demonstrate the presence of the tumour, CTA confirms its hypervascular nature, delineates its arterial supply (for example, feeders from the ascending pharyngeal or occipital arteries) and assesses involvement of adjacent structures such as the jugular bulb or carotid artery. Moreover, in a single acquisition, CTA can demonstrate the tumour blush and map arterial feeders – information indispensable when planning endovascular embolisation or surgical resection.^14^

CT angiography is also valuable for identifying rare vascular malformations causing CHL. Congenital anomalies such as an aberrant internal carotid artery or a persistent stapedial artery in the middle ear can present with pulsatile tinnitus and CHL. High-resolution CT may show an unusual tubular structure or dehiscent bony canal, but CTA definitively identifies the vessel and outlines its course. Likewise, a high-riding or dehiscent jugular bulb can encroach upon the middle ear space; CTA reveals venous anatomy and any relevant variants. In many cases, these diagnoses can be sufficiently established on non-contrast CT, which shows absent bone or abnormal canals, and contrast-enhanced imaging is reserved for surgical planning– for example, to distinguish a dehiscent jugular bulb (venous) from a glomus tumour (arterial).^37^

### CT angiography in the spotlight: Performance unleashed

When applied judiciously, CTA excels at depicting vascular lesions. It reliably reveals glomus tumours as avidly enhancing masses with enlarged feeding arteries. Its sensitivity for detecting aberrant vessels is equally high – modern thin-slice protocols clearly display any artery following an unusual course. Specificity is also excellent, as non-vascular masses neither enhance strongly nor connect to known arteries.

Because CTA is reserved for select cases, broad sensitivity and specificity statistics for ‘CTA in CHL’ are not well established. Nevertheless, CTA occupies a niche but pivotal role: providing diagnostic confirmation and detailed surgical roadmaps for vascular middle ear pathologies causing conductive hearing impairment. In all other cases, its routine use is not justified. Thus, clinicians typically perform CTA only after CT or MRI suggests a vascular anomaly requiring further clarification.^4^

## Age-specific considerations: Paediatric versus adult patients

The causes of CHL differ between paediatric and adult populations, influencing imaging strategies. In children, CHL often results from congenital abnormalities or sequelae of chronic otitis media. Common findings include ossicular malformations (e.g. stapes fixation, absent incus), EAC atresia or syndromic deformities such as Treacher Collins or CHARGE syndrome. These conditions rely primarily on CT for surgical planning, as most anomalies involve bony structures that are well visualised on imaging. Studies show that CT frequently identifies aetiologies such as ossicular dysplasia, fixation or malformation. Congenital cholesteatoma may also occur behind an intact tympanic membrane, typically presenting as a pearly white anterosuperior mass causing unilateral CHL with a normal drum.^38^ High-resolution CT (≤ 0.5 mm) or cone-beam CT is used to map the precise quadrant of involvement, detect ossicular erosion and assess mastoid extension. Non-echo-planar diffusion-weighted MRI provides essential soft tissue contrast, reliably distinguishing cholesteatoma from effusion or granulation tissue. It is now the modality of choice for both preoperative delineation and detection of residual disease as small as 3 mm – 5 mm. Congenital cholesteatoma can also be staged with the four-tier Potsic classification, which correlates closely with residual disease risk, guiding operative planning and postoperative surveillance.^39^

In adults, CHL is most often acquired. Otosclerosis typically arises in young to middle-aged adults and is best detected with submillimetre HRCT, which demonstrates high diagnostic accuracy for fenestral or cochlear foci of demineralisation.^40^ Chronic otitis media and cholesteatoma often begin in adolescence and persist into adulthood. Ossicular disruption following trauma peaks in young adults. Highly vascular glomus tympanicum or jugulare paragangliomas usually present in the fifth to sixth decades with pulsatile tinnitus and a retrotympanic mass.^41^ ‘Third-window’ defects, such as SSC dehiscence, although congenital, typically become symptomatic later in adulthood.^2^

Because younger patients carry a higher lifetime risk of radiation–induced malignancy, any unexplained – or especially unilateral – adult conductive deficit not clearly attributable to otitis is routinely investigated first with HRCT to screen for otosclerosis, ossicular discontinuity, third-window lesions or other structural causes. MRI is then added selectively to characterise soft tissue masses, map cholesteatoma extent or delineate vascular tumours, complementing rather than replacing CT in the diagnostic work-up.

### Tailored imaging modality selection

In general, CT is the first-line modality for both adults and children when an osseous cause of CHL is suspected. However, extra caution is exercised in paediatric imaging because of radiation exposure. Paediatric CT protocols use the lowest feasible dose that maintains diagnostic quality, as children are more sensitive to ionising radiation and have a longer post-exposure lifespan. Current low-dose paediatric CT protocols maintain an effective dose of approximately 0.03 mSv – 0.08 mSv – representing a 70% – 80% reduction compared with standard adult temporal-bone scans.^42^ Radiologists strictly adhere to the ALARA (as low as reasonably achievable) principle, as promoted by Image Gently®, when performing paediatric temporal bone CT. Strategies include reducing tube current, limiting scan length to the temporal bones and avoiding unnecessary bilateral scans if the problem is clearly unilateral (unless evaluation of the contralateral side is required for surgical planning).

MRI in children often requires sedation because the scan duration and the requirement to remain motionless are challenging. Sedation or general anaesthesia carries its own risks: prospective studies report that approximately 3% of children experience brief hypoxemia during sedation, and sedation is unsuccessful in up to 7% of cases.^43^ Therefore, the decision to perform MRI in a child with hearing loss is carefully weighed. If CT has already provided sufficient information, such as identifying a clear ossicular malformation, many clinicians defer MRI in young children. Conversely, MRI is crucial when there is a sensorineural component or suspicion of cochlear nerve pathology, for example, during evaluation for cochlear implantation. In older children and adolescents, MRI can often be performed without sedation, similar to adult patients.

## Comparison of imaging modalities by condition

Table 1 summarises the roles of CT, MRI and CTA in diagnosing common causes of CHL, highlighting the preferred imaging modality for each condition and outlining the unique contributions and advantages of each technique.

**TABLE 1:** Comparison of imaging modalities by condition.

Condition/aetiology	HRCT	HR-MRI	CTA/MRA
Otosclerosis (stapedial fixation)	First-line: fissula ante fenestram lucency and/or oval-window thickening; flags pericochlear halo (halo sign)	Second-line (limited role); poor bone detail; shows marrow/cochlear enhancement only in active extensive disease; usually normal in fenestral; use for mixed CHL-SNHL or neural concerns	Not applicable
Chronic otitis media with cholesteatoma	First-line: middle-ear and/or mastoid soft-tissue mass and bony erosions (scutum, ossicles, tegmen); high sensitivity/NPV but low specificity; maps extent and fistulae for surgery	Non-echo-planar DWI confirms cholesteatoma (~90% – 94%); Gd MRI maps spread/complications; key for pre- and post-op assessment; may avert second-look surgery	Only if vascular tumour suspected (e.g. glomus)
Ossicular chain discontinuity (trauma or erosion)	Gold standard: detects ossicular breaks and/or dislocations and temporal-bone fractures; maps intact ossicles for ossiculoplasty	Minimal: shows fluid and/or blood only; poor ossicle detail – reserve for suspected inner-ear and/or nerve injury	Not applicable
Congenital ossicular anomalies (± external auditory canal atresia)	First-line: charts congenital ear anatomy – ossicular absence and/or malformation or fixation, facial-nerve course, middle-ear size; feeds Jahrsdoerfer atresia grading and flags inner-ear anomalies	Adjunct: evaluates cochlear nerve and inner ear in microtia and/or atresia or syndromic and/or mixed loss; otherwise, CT is sufficient	Not applicable
Temporal bone trauma (fractures)	Primary and/or emergency: classifies fractures (longitudinal, transverse, otic-capsule), detects ossicular disruption and/or haemotympanum; guides surgery (facial-nerve decompression)	Secondary: for neuro or inner-ear complications – shows labyrinth and/or nerve injury; unnecessary for isolated ossicular CHL	Only if vascular injury is suspected
Glomus tumours (paragangliomas: e.g. glomus tympanicum and/or jugulare)	Second-line: middle-ear and/or jugular mass with bony erosion suggests glomus; cannot confirm vascularity	Essential: contrast ‘salt-and-pepper’ mass; delineates brain and/or neck spread and bilateral lesions; distinguishes from high jugular bulb	Maps feeders for embolisation, confirms pulsatile paraganglioma; mainly for pre-op planning in larger tumours
‘Third window’ syndrome (e.g. superior semicircular canal dehiscence)	Canal-plane reconstruction shows SSCD roof defect and size; also flags EVAS (mixed hearing loss)	Not applicable	Not applicable

HRCT, high-resolution computed tomography; HR-MRI, high-resolution magnetic resonance imaging; CTA, computed tomography angiography; CHL, conductive hearing loss; SNHL, sensorineural hearing loss; Gd MRI, gadolinium-enhanced magnetic resonance imaging; SSCD, superior semicircular canal dehiscence; EVAS, enlarged vestibular aqueduct syndrome.

As shown, CT and MRI are complementary. CT excels at depicting bony anatomy and serves as the primary tool for the initial diagnosis of CHL, while MRI is superior for characterising soft tissue lesions and detecting complications. CT angiography is used selectively, mainly to illuminate vascular lesions. Understanding these distinctions enables clinicians to select the most appropriate modality at each stage of the CHL work-up.

## Conclusion

High-resolution CT of the temporal bone remains the primary imaging modality for evaluating CHL because of its submillimetre resolution, which allows detailed visualisation of bony structures – from stapes fixation in otosclerosis to ossicular discontinuities and complex congenital malformations. MRI serves as an essential complementary tool for soft-tissue characterisation: non-echo-planar diffusion-weighted and contrast-enhanced sequences facilitate detection or exclusion of cholesteatoma, delineation of tumours and inflammatory disease and evaluation of sensorineural components in mixed hearing loss cases. Although not routinely performed, CTA plays a valuable role in assessing suspected vascular middle ear lesions by revealing feeding vessels and their anatomic relationships.

In paediatric patients, imaging protocols emphasise radiation dose reduction, reserving CT for cases where findings will impact clinical management. In adults, a broader differential diagnosis and lower lifetime radiation risk support more frequent CT use, with MRI or CTA added selectively for soft-tissue or vascular clarification. Current appropriateness guidelines recommend temporal bone CT as the first-line modality, with MRI or CTA reserved for further evaluation as indicated.

Looking forward, emerging technologies such as ultra-HRCT and advanced non-echo-planar DWI (e.g. PROPELLER) promise improved detection of millimetre-scale lesions. Experimental CT–MRI fusion techniques may further enhance diagnostic specificity and surgical planning. With continued advancements and optimised multimodal imaging protocols, clinicians can anticipate even greater accuracy and confidence in diagnosing the full spectrum of CHL aetiologies in daily practice.
